# Childhood Systemic Lupus Erythematosus: Clinical and Immunologic Patterns in Mexican Children

**DOI:** 10.7759/cureus.59851

**Published:** 2024-05-07

**Authors:** Edgar E Morales Montes, Iris P García Herrera, Yesenia Hernández Torres, Linda F Perez Perez, Luis A Aparicio Vera

**Affiliations:** 1 Pediatrics, Hospital para el Niño Poblano, Puebla, MEX; 2 Pediatric Cardiology, Hospital para el Niño Poblano, Puebla, MEX; 3 Pediatric Rheumatology, Hospital para el Niño Poblano, Puebla, MEX

**Keywords:** mexican, pediatric, sex differences, male lupus, gender, lupus erythematosus

## Abstract

Introduction

Systemic lupus erythematosus (SLE) is a complex autoimmune disease with heterogeneous clinical and laboratory features. The incidence increases markedly in women. The reason for the predominance of the female gender is still under study. The ethnicity could influence the clinical and serological features of SLE.

Material and methods

This is a retrospective, descriptive, and longitudinal study. We studied 89 patients diagnosed with childhood-onset systemic lupus erythematosus (cSLE) in our center in 2000-2020 for men and 2010-2020 for women. We investigated disease manifestations, ranging from clinical symptoms to renal involvement, at the time of diagnosis and compared them by gender.

Results

We studied 89 patients, comprising 23 males and 66 females. The mean age for both groups was 12 years. Concerning clinical manifestations, serositis exhibited a higher prevalence among males, while hair loss was more prominent among females. In the paraclinical evaluation, noteworthy differences were observed regarding average hemoglobin levels and the prevalence of positive anti-DNA antibodies. Males demonstrated an average hemoglobin level of 11.47 g/dL, whereas females displayed 9.84 g/dL (p=0.017). The prevalence of anti-DNA antibodies exhibited marked elevation in males, at 88.9%, compared to females' 42.9% (p=0.024). On a contrary note, our male cohort displayed heightened prevalence in using hydroxychloroquine, cyclophosphamide, and mycophenolate.

Similarly, renal involvement presented a higher prevalence in males (100% against 83.3%), albeit lacking statistical significance. Nevertheless, significant disparities emerged in the occurrence of granular casts, proteinuria, and the average glomerular filtration rate, with males experiencing greater impact in each instance. Finally, it is noteworthy that the application of mycophenolate and azathioprine was more frequently observed among patients with renal involvement.

Conclusion

cSLE patients in our inception cohort showed statistical significance in dermatological and vascular manifestations, serositis, granular casts, low kidney glomerular filtration, and high proteinuria, which are predominant in male patients. Immunological features were predominantly positive in ds-DNA antibodies for male patients. Separation by gender for future studies might identify a better treatment strategy.

## Introduction

Systemic lupus erythematosus (SLE) is a complex autoimmune disease with different clinical and laboratory features, variable courses, and prognoses. It involves almost all organs and systems, including skin, joints, kidneys, brain, lungs, and heart. The incidence of SLE is markedly increased in females, with an incidence reported to be 6-10 times higher in women than in men and more prevalent in women of childbearing age. The prevalence of SLE has been estimated at 30-50 per 100,000, which equates to approximately 500,000 patients in Europe and 250,000 in the United States [1].

The diagnosis of SLE is based on a set of clinical and immunological manifestations. Eager to unify appropriate classification criteria, multiple classifications have been created. In this present study, two of these classifications have been used. The first is the American College of Rheumatology (ACR) classification criteria, which require at least 4 of 11 elements to be met. The second one is the Systemic Lupus International Collaborating Clinics (SLICC) 2012 classification, which is based on 11 clinical criteria and six immunological criteria, whereby at least four elements must be met, with at least one clinical and one immunological criterion, or there must be the presence of lupus nephritis with positive ANA or anti-dsDNA. The European League Against Rheumatism (EULAR)/ACR classification has been recently published, but it was not considered for this study [2].

It has been described as a worse prognosis with more severe affectation in some studies of SLE in male pediatric populations. Ancestry, race, and ethnicity appear to impact SLE manifestations and severity. Hispanic patients tend to develop lupus earlier and have more severe and more active disease. In our study, we seek to identify and describe the clinical and immunological characteristics of the Mexican pediatric SLE population and compare them to those of other populations described.

## Materials and methods

Patients in the study

The present study is retrospective, descriptive, and longitudinal. We selected patients who met the diagnostic criteria for childhood-onset systemic lupus erythematosus (cSLE) based on the classification criteria of either ACR or SLICC during their initial evaluation at the pediatric rheumatology service of Hospital para el Niño Poblano.

For patient selection, we considered the period from 2000-2020 for males and 2010-2020 for females. The time frame was determined based on the prevalence of the diagnosis in both groups and sample size considerations. No exclusion criteria were applied.

Approval for the study protocol was obtained from our center's ethical committee.

Data extraction

The clinical characteristics considered in this study included arthritis, arthralgias, myalgias, hair loss, fatigue, fever, oral ulcers, asthenia and adynamia, weight loss, malar erythema, discoid lupus, serositis, central nervous system alterations, and cardiovascular disorders.

Serological markers considered were hemoglobin, leukocytes, lymphocytes, platelets, direct Coombs test, albumin, cholesterol, triglycerides, complement C3 and C4, CH-50, IgG, antinuclear antibodies, anti-La antibody, anti-Ro antibody, anti-double-stranded DNA (anti-dsDNA) antibody, anti-RNP antibody, anti-Sm antibody, rheumatoid factor, anti-histones antibody, anti-cardiolipin antibodies, anti-beta-2-glycoprotein antibodies, and lupus anticoagulant.

The nephrological assessment encompassed urinalysis findings such as granular casts, hematuria, and proteinuria; the presence of nephrotic or nephritic syndrome; estimated glomerular filtration rate (calculated using the modified Schwartz formula, and Chronic Kidney Disease Epidemiology Collaboration (CKD-EPI) for one teenager whose height was not recorded in their medical records); undergoing renal biopsy; histopathological diagnosis; compromised renal function (acute kidney injury (AKI) or chronic kidney disease (CKD)) with no other suspected etiology; and finally, the requirement of renal substitution therapy within the first month of diagnosis. Significant proteinuria was defined as a single sample equal to or greater than 10 mg/dL or a 24-hour collection equal to or exceeding 4 mg/m²/hr. Nephrotic range proteinuria was defined as a single sample equal to or exceeding 300 mg/dL or a 24-hour collection equal to or surpassing 40 mg/m²/hr. As for biopsy range proteinuria, it was defined as exceeding 1 g within a 24-hour quantification. This distinction was made due to the absence of 24-hour collections for all patients at diagnosis.

The biopsies were classified according to the World Health Organization and the International Society of Nephrology/Renal Pathology Society (ISN-RPS). For the statistical analysis, renal biopsies were categorized into three groups: "LN 1-2" (Lupus Nephritis Class 1- 2), "LN 3-5" (Lupus Nephritis Class 3-5), and "Others," referring to those who did not meet the criteria for the previous grouping according to ISN-RPS classification. 

To define renal involvement, we considered the presence of one or more of the following: granular casts, hematuria, proteinuria, glomerular syndrome, CKD, or AKI at diagnosis.

The treatments considered in this study were methylprednisolone, prednisone, methotrexate, azathioprine, cyclophosphamide, cyclosporine, mycophenolate, hydroxychloroquine, and chloroquine.

Statistical analysis

Statistical analysis was performed by comparing each variable by gender. Student's t-test and Mann-Whitney U test were used for parametric and nonparametric distributions, respectively, to analyze continuous variables. For categorical data, we used the Chi-square test, and in cases where any of the cells had an expected count of fewer than 5, Fisher's exact test was applied. Data were analyzed using SPSS Statistics 25. A p-value less than 0.05 was considered statistically significant in all analyses.

## Results

Initial evaluation

Eighty-nine patients were evaluated, including 23 males and 66 females. Due to the study's retrospective nature, not all patients had complete assessments. The mean age for males was 12.2 years, and for females, 12.7 years, with no significant difference between the two groups (p=0.292) (Table 1).

**Table 1:** Initial evaluation Quantitative variables were assessed using the Student’s t-test, whereas qualitative variables were evaluated using the Chi-square test and Fisher’s exact test; p values < 0.05 were considered statistically significant. Variables that did not appear in either of the two groups are denoted with a “-“ and were not subjected to statistical analysis.

	Male	Female	P*
Age			
Age (mean ± SD, years)	12.26 ± 2.1	12.73 ± 2.9	0.292
Clinical manifestations			
Arthritis	30.4% (7/23)	37.9% (25/66)	0.522
Arthralgias	52.2% (12/23)	45.5% (30/66)	0.578
Myalgias	13% (3/23)	10.6% (7/66)	0.714
Hair loss	4.3% (1/23)	27.3% (18/66)	0.020*
Oral ulcers	8.7% (2/23)	13.6% (9/66)	0.721
Malar rash	43.5% (10/23)	43.9% (29/66)	0.969
Photosensitivity	-	6.1% (4/66)	-
Discoid lupus	8.7% (2/23)	12.1% (8/66)	1.000
Raynaud’s phenomenon	4.3% (1/23)	4.5% (3/66)	1.000
Fatigue	8.7% (2/23)	6.1% (4/66)	0.646
Asthenia and adynamia	26.1% (6/23)	34.8% (23/66)	0.440
Weight loss	30.4% (7/23)	21.2% (14/66)	0.370
Fever	39.1% (9/23)	37.9% (25/66)	0.915
Purpura	8.7% (2/23)	10.6% (7/66)	1.000
Thrombosis	4.3% (1/23)	7.6% (5/66)	1.000
Pleural effusion	21.7% (5/23)	21.2% (14/66)	1.000
Serositis	47.8% (11/23)	19.7% (13/66)	0.009*
Alveolar hemorrhage	-	1.5% (1/66)	-
Cardiac compromise	17.4% (4/23)	13.6% (9/66)	0.151
Central nervous system disorders	13% (3/23)	30.3% (20/66)	0.103
Laboratories			
Hemoglobin (g/dL)	11.47 ± 2.32	9.84 ± 2.91	0.017*
Leukocytes (10^3/µl)	7.33 ± 6.22	6.49 ± 3.94	0.783
Lymphocytes (10^3/µl)	1.25 ± 1.46	1.45 ± 9.36	0.300
Platelets (10^3/µl)	239.35 ± 109.16	236.69 ± 165.53	0.943
Albumin (g/dL)	2.47 ± 0.8	2.98 ± 0.89	0.077
Cholesterol (mg/dL)	193.94 ± 85.12	172.11 ± 91.22	0.313
Triglycerides (mg/dL)	239.38 ± 135.27	232.94 ± 159.85	0.703
Coombs	60% (6/10)	49% (25/51)	0.731
CH50	57.1% (4/7)	81.8% (9/11)	0.326
High IgG levels	33.3% (3/9)	36.4% (16/44)	0.057
ANAS	100% (17/17)	94.9% (56/59)	1.000
Homogenous	57.1% (8/14)	48% (12/25)	0.584
Speckled	21.4% (3/14)	32% (8/25)	0.713
Centromere	7.1% (1/14)	8% (2/25)	1.000
Nucleolar	14.3% (2/14)	8% (2/25)	0.609
Anti-La	-	6.3% (2/32)	-
Anti-Ro	50% (2/4)	41.2% (14/34)	1.000
Anti-DNA	88.9% (8/9)	42.9% (18/42)	0.024*
Anti-RNP	16.7% (2/12)	39% (16/41)	0.185
Anti-SM	25% (4/16)	37% (17/46)	0.384
Anti SCL70	-	50% (1/2)	-
Rheumatoid factor	50% (2/4)	22.6% (7/31)	0.268
Anti-cardiolipin	20% (1/5)	27% (10/37)	1.000
Anti-ß2	25% (1/4)	11.8% (4//34)	0.446
Lupus anticoagulant	60% (3/5)	40% (14/35)	0.634
VDRL	25% (1/4)	9.8% (5/51)	0.379
Initial treatment			
Methylprednisolone	69.6% (16/23)	47% (32/66)	-
Prednisone	73.9% (17/23)	68.2% (45/66)	-
Deflazacort	8.7% (2/23)	3% (2/66)	-
Betamethasone	8.7% (2/23)	-	-
Hydroxychloroquine	78.3% (18/23)	54.5% (36/66)	-
Leflunomide	-	1.5% (1/66)	-
Chloroquine	8.7% (2/23)	3% (2/66)	-
Sulfazalazine	-	3% (2/66)	-
Methotrexate	34.8% (8/23)	18.2% (12/66)	-
Azathioprine	13% (3/23)	21.2% (14/66)	-
Enoxaparin	4.3% (1/23)	4.5% (3/66)	-
Immunoglobulin	4.3% (1/23)	4.5% (3/66)	-
Plasmapheresis	-	4.5% (3/66)	-
Rituximab	-	1.5% (1/66)	-
Cyclophosphamide	60.9% (14/23)	12.1% (8/66)	-
Cyclosporine	4.3% (1/23)	-	-
Mycophenolate	56.5% (13/23)	28.8% (19/66)	-

Clinical Manifestations

Clinical manifestations at the time of diagnosis were compared between males and females (Table 1). Musculoskeletal involvement and the presence of arthritis, arthralgias, and myalgias at diagnosis were evaluated, but no significant difference was found in their prevalence between males and females. Regarding dermatological and vascular manifestations such as hair loss, oral ulcers, malar rash, photosensitivity, discoid lupus, and Raynaud's phenomenon, only hair loss showed a significant difference, with a prevalence of 4.3% (1/23) in males and 27.3% (18/66) in females (p=0.020). Constitutional symptoms, including fever, fatigue, weight loss, asthenia, and adynamia, did not show significant differences between the two groups. Hematological manifestations, such as purpura and thrombosis, were assessed, but no significant difference was observed. The presence of serositis and pleural effusion was also evaluated, with serositis being significantly more prevalent in males (47.8%; 11/23) compared to females (19.7%; 13/66) (p=0.009). 

Finally, the prevalence of cardiac involvement, alveolar hemorrhage, and neurological manifestations was compared, but no significant difference was found. Of note is that in our sample, photosensitivity and alveolar hemorrhage were observed exclusively in female individuals.

Paraclinical Evaluation

The prevalence of major paraclinical abnormalities at the time of diagnosis was compared between males and females, as shown in Table 1. Hematological parameters, including hemoglobin levels, leukocyte count, and platelet count, were compared, and a significant difference was found in hemoglobin levels, with a mean of 11.47g/dL in males and 9.84g/dL in females (p=0.017). No significant difference was found in the chemistry panel, which included albumin, cholesterol, and triglycerides. Immunological evaluation included Coombs test, CH50, IgG levels, ANA (and their respective patterns), anti-La, anti-Ro, anti-DNA, anti-RNP, anti-SM, anti-SCL70, rheumatoid factor, anti-cardiolipins, anti-β2, lupus anticoagulant, and VDRL; only the prevalence of anti-DNA antibodies showed a significant difference, with a prevalence of 88.9% in males and 42.9% in females (p=0.024). Notably, our study population encompasses three patients with negative antinuclear antibodies (ANA), each exhibiting distinct phenotypes. Patient 1 presents with serositis, hematologic disease, low complement levels, and proteinuria without kidney biopsy (classified by SLICC). Patient 2 displays renal disease with a kidney biopsy, serositis, hematologic disease, and a positive Coombs test (classified by SLIC). In contrast, patient 3 demonstrates renal disease with a kidney biopsy, positive anti-DNA antibodies, and hematologic abnormalities. No males exhibiting positive anti-SCL70 or anti-LA antibodies were encountered during our review.

Initial Treatment

The treatments administered at the time of diagnosis were compared between males and females, as reported in Table 1. The treatments used included methylprednisolone, prednisone, deflazacort, betamethasone, hydroxychloroquine, leflunomide, chloroquine, sulfasalazine, methotrexate, azathioprine, enoxaparin, immunoglobulin, plasmapheresis, rituximab, cyclophosphamide, cyclosporine, and mycophenolate. Differences were found in the use of hydroxychloroquine, with a usage rate of 78.3% (18/23) in males and 54.5% (36/66) in females; cyclophosphamide, with a usage rate of 60.9% (14/23) in males and 12.1% (8/66) in females; and mycophenolate, with a usage rate of 56.5% (13/23) in males and 28.8% (19/66) in females. Leflunomide, sulfasalazine, plasmapheresis, and rituximab were not employed within the male population, whereas betamethasone and cyclosporine were not utilized within the female population.

Renal assessment

Renal involvement was assessed by comparing the presence of glomerular syndromes, biopsy results, urinalysis findings, renal function, the prevalence of acute kidney injury or chronic kidney disease, and the need for renal replacement therapy between males and females (Table 2). When comparing renal involvement, a higher prevalence was observed in males, although it was not statistically significant (p=0.060). The prevalence of nephrotic, nephritic, and nephrotic-nephritic syndromes at diagnosis showed no difference between males and females. Regarding the prevalence of renal biopsies, 60.9% (14/23) of males underwent a biopsy, compared to 28.8% (19/66) of females. In the context of biopsy results, all male subjects displayed patterns compatible with Lupus Nephritis Class 3-5, whereas the female subjects demonstrated a uniform distribution spanning Lupus Nephritis Class 1-2 and Class 3-5. Three biopsies were designated as unclassifiable within the aforementioned groups, encompassing crescentic glomerulonephritis, thrombotic microangiopathy, and podocytopathy. Urinalysis findings, including the presence of granular casts, proteinuria, and hematuria at diagnosis, showed significant differences in the first two parameters. The prevalence of granular casts was higher in males (65.2%; 15/23) compared to females (34.9%; 22/63) (p=0.012). Regarding proteinuria, males had a prevalence of 95.7% (22/23), whereas females had a prevalence of 68.3% (43/63) (p=0.000). When considering just proteinuria in the nephrotic range, the prevalence in males was 52.2% (12/23), and in females, it was 28.2% (18/63) (p=0.042). In terms of proteinuria indicating the necessity of kidney biopsy by a 24-hour urine collection test, a higher prevalence was observed in males (68.8%; 11/16) compared to females (51.4%; 18/35), but it was not statistically significant (p=0.362). Regarding renal function, the mean estimated glomerular filtration rate was significantly lower in males (64.1±44.5 ml/min/1.73m^2^) compared to females (102.6±51.8 ml/min/1.73m^2^) (p=0.003). No significant differences were found in the prevalence of AKI, CKD, or the need for renal replacement therapy during the first month after diagnosis.

**Table 2:** Renal Assessment Quantitative variables were assessed using the Student’s t-test, whereas distribution of absolute (and relative) frequencies were evaluated by the Chi-square test and Fisher’s exact test; p values < 0.05 were considered statistically significant. ✦Estimated glomerular filtration rate was calculated using the Schwartz formula. Variables that did not appear in either of the two groups are denoted with a “-“ and were not subjected to statistical analysis.

	Male	Female	P*
Age			
Age (mean ± SD, years)	12.26 ± 2.1	12.73 ± 2.9	0.292
Renal involvement			
	100% (23/23)	83.3% (55/66)	0.060
Glomerular syndromes			
Nephrotic	21.7% (5/23)	12.7% (8/63)	0.320
Nephritic	8.7% (2/23)	3.2% (2/63)	0.289
Mixed	17.4% (4/23)	6.3% (4/63)	0.202
Renal biopsy			
Biopsy	60.9% (14/23)	28.8% (19/66)	-
NL1–2	-	42.1% (8/19)	-
NL3–5	100% (14/14)	42.1% (8/19)	-
Others	-	15.7% (3/19)	-
Urinalysis			
Sediment			
Granular casts	65.2% (15/23)	34.9% (22/63)	0.012*
Proteinuria			
Proteinuria	95.7% (22/23)	68.3% (43/63)	0.009*
Nephrotic range	52.2% (12/23)	28.2% (18/63)	0.042*
Biopsy range	68.8% (11/16)	51.4% (18/35)	0.362
Hematuria			
Hematuria	95.7% (22/23)	78.1% (50/64)	0.103
Renal function			
EGFR✦ (mean ± SD)	64.1 ± 44.5	102.6 ± 51.8	0.003*
Kidney injury			
Acute kidney injury	31.8% (7/22)	20.6% (13/63)	0.381
Chronic kidney disease	36.4% (8/22)	27% (17/64)	0.406
Replacement therapy			
Peritoneal dialysis	8.7% (2/23)	3% (2/67)	0.274
Hemodialysis	-	4.5% (3/66)	0.566

## Discussion

Systemic lupus erythematosus is a clinically heterogeneous autoimmune disease of unknown etiology. Compared to adult-onset SLE, cSLE is more aggressive, with higher disease activity and medication burden that contributes to the increased morbidity and mortality associated with the disease, more severe organ manifestations, the presence of greater damage at the time of diagnosis, and a higher incidence of renal, cardiovascular, and neuropsychiatric involvement [3-5]. In SLE, the male sex has also been associated with more severe forms of the disease in terms of clinical manifestations and prognosis, with renal involvement and serological manifestations such as hypocomplementemia and anti-dsDNA autoantibodies reported as more common in male patients, although there are some inconsistencies about the type of differences in several studies [6].

When we compared male and female patients at our hospital, the predominant features of our male population were serositis and renal involvement (granular casts, high proteinuria, and low glomerular filtration rate), as in the study by Front and Cervera [7]. They studied 261 patients with SLE at the Hospital Clinic of Barcelona between 1980 and 1990; 12% were men. They included some pediatric patients with SLE. The assessment included clinical and serological characteristics. They found that discoid lupus lesions and serositis more often presented at the onset of the disease, with no other clinical features relevant, because there was no statistical significance. Tan et al. found similar results concerning malar rash and arthralgias and reported more renal involvement, thrombotic events, and hypertension [1].

Recently, in 2019, Ramírez-Sepulveda found some gender-related differences. In their study, 166 male patients (mean age of 36 +/- 15 years old) were significantly more often affected by serositis and renal disorders such as proteinuria [8]. However, the entire population considered in the study was older than 18 years. Other reports from around the world suggested renal involvement predominance, older age at disease onset, and more prevalence in serositis, seizures, and discoid lupus in SLE in males [9-13]. Dzifa Dey et al. reported serositis and constitutional features as the most common features in 13 males from Ghana. However, this report does not include pediatric patients, and they did not compare men with women [4]. Our cSLE male patients had more serositis and proteinuria but not seizures and discoid lupus. The Grupo Latino de Estudio del Lupus (GLADEL) reported on 123 males from México, Peru, Venezuela, Argentina, Brazil, Colombia, Chile, and Guatemala, showing constitutional symptoms and a high prevalence of neurological manifestations. The authors evaluated the influence of the male gender in an inception cohort of Latin-American patients. They reported a predominance of fever and weight loss (42.3% and 23.6%, respectively) [14]. Molina et al. found similar results with 107 patients from Columbia and México with a high prevalence of skin disease and arthritis as well [15].

Renal disease has been described as more aggressive in males and children with SLE. There are some Hispanic and Latin-American reports, such as the Lupus in Minorities, Nature versus Nurture (LUMINA) cohort, with equal numbers of Hispanic, African, American, and Caucasian patients. The researchers included 63 males, reported a 63.5% renal involvement, and identified male gender as a risk factor for accelerated damage [16]. In North America, based on the Hopkins Lupus Cohort, male patients were identified with a greater incidence of renal, neuropsychiatric, and cardiovascular disease [17].

Studies on children are more limited; Ali-Mayouf and Sonbul reported on 13 male Saudi children. Logistic regression analysis showed a significant association of high SLICC/ACR score with early onset disease and male gender, and renal disease requiring dialysis and renal transplant was associated with male gender [18].

The renal disorder at our hospital was significant for casts, high proteinuria, and low glomerular filtration rate. However, our patients did not need more therapy with dialysis or hemodialysis. We consider that high proteinuria and low glomerular filtration rate could be the rationale for preferring mycophenolate mofetil as treatment. Surprisingly, there are no significant differences between CKD and AKI. Renal abnormalities have been described as more common among individuals of African, Asian, or Mediterranean descent [19]. However, our male patients exhibited renal features at onset, representing a 100% incidence rate in this demographic. This notable percentage warrants consideration in cases of pediatric lupus debuting in Mexico. Notably, the renal disorder at onset was not overtly aggressive, underscoring the potential impact of early diagnosis and treatment.

In delineating treatment approaches, it becomes evident that factors such as the study's temporal framework and institutional resources warrant careful consideration. We provide a breakdown of treatment distribution owing to the lack of documented differences in initial management between male and female cSLE patients in Mexico. However, we advocate for additional investigations to elucidate the potential influence of other variables beyond clinical presentation on the initial therapeutic strategies employed in these cases.

In Europe, a higher prevalence of thromboses and antiphospholipid syndrome in male patients has been reported [20]. Among our patients, these features were infrequent.

While other features reported to occur more frequently in male systemic lupus, such as neurological or discoid lupus, did not demonstrate statistical significance in our study, our pediatric Mexican population did exhibit a higher incidence of serositis and renal disease, consistent with other findings. Conversely, female patients displayed statistical significance in hemoglobin levels and hair loss.

The serological profile reported is similar to that observed in our study, with anti-dc-DNA predominance in males [4,7,21]. However, other groups reported no significant difference between men and women while considering antinuclear antibodies, double-stranded DNA, antiphospholipid antibodies, and antibodies to extractable nuclear antigens and complement.

It is pertinent to note that our population exhibits a 3.9% rate of negative antinuclear antibodies, with three patients demonstrating diverse phenotypes. Patient 1 exhibits serositis, hematologic disease, low complement levels, and proteinuria without kidney biopsy (Classified by SLICC). Patient 2 presents renal disease with a kidney biopsy, serositis, hematologic disease, and positive Coombs test (Classified by SLICC), while patient 3 displays renal disease with a kidney biopsy, positive anti-DNA antibodies, and hematologic abnormalities. Although antinuclear antibody testing remains pivotal in diagnosis, other clinical and laboratory features contribute to the classification of lupus. One potential explanation for the negative results is the methodology employed; nonetheless, it's worth noting that the prevalence of antinuclear antibody-negative lupus has been estimated to range from 5-10%[22].

There are currently no reports similar to these findings within the Mexican population. While some reports do include causes of death among young people with lupus, there is a lack of detailed descriptions regarding the clinical or serological features of these patients [23, 24]. Regarding pediatric Mexican patients, only one report from the Instituto Nacional de Pediatria exists, which includes 159 pediatric patients with lupus, comprising 105 females and 54 males. In this study, multivariate analysis revealed that the presence of proteinuria and abnormal electroencephalograms were independent factors associated with mortality [25]. However, it is important to note a couple of significant points. Firstly, our report encompasses all pediatric lupus features, both serological and clinical. Secondly, we highlight the primary phenotype at the onset.

The pathophysiological mechanism responsible for the sexual dimorphism is still unclear. Many factors have been considered. Some genetic differences have been described, such as the T cell DNA methylation that could contribute to female susceptibility to lupus. In this way, an apparent high incidence of lupus in men with Klinefelter's syndrome seems to reinforce this hypothesis [26]. Other genetic alterations have been related to toll-like receptors [27].

Autoimmune diseases that are more frequent in men are described with more acute inflammation, with a typical Th1 immune response. In contrast, autoimmune diseases that are more frequent in women are associated with antibody-mediated pathology and Th2-mediated immune response [11].

The hormonal approach has been implicated with the sex differences; however, the studies have not been conclusive. The sex hormones have been shown to have an important action in the immune system, including the B and T cells, dendritic cells, and cytokine networks. The female hormonal influences could support the survival of autoreactive B cells, whereas male hormones produce the opposite [4]. An estrogen effect has been suggested. In children, the hormonal effects are presumably minimal; thus, female-to-male differences should be minimal. The primary effects of estrogen are mediated by estrogen receptors (ERs). ER α is expressed in most immune cells and has been related to Th2 immune response, and enhancesinterferon-γ, TNFα, TGFβ, interleukin-1, interleukin-4, and interleukin-5 [25]. Some researchers have reported a normal level of estrogen in male and female patients with SLE; however, the estrogen metabolism causes the difference between genders. The molecular marker of estrogen action may be calcineurin, and these days, calcineurin inhibitors are some of the useful therapies for SLE [28].

Other related hormones are the androgens and prolactin, the first of which has been found in lower plasma levels in female patients with SLE, and both male and female mice have been reported as having enhanced adrenal and immune responses to endotoxin after gonadectomy. Also, prolactin has been related to lupus flare disease activity in children [28-30].

Considering the associated morbidities in our group of patients, only the diagnosis of rhabdomyosarcoma was reported in one of the patients. No association with Sjögren Syndrome or other rheumatologic diseases was documented, as other researchers have described [13].

A novel clinical criterion in the EULAR ACR classification-unexplained fever-is relatively common. Among our pediatric patients, 90% have this clinical feature.

The limitations of our study stem from its retrospective design and the absence of certain data in our records. A prospective study is needed to ascertain the subsequent evolution and identify prognostic factors associated with surveillance.

## Conclusions

Childhood-onset systemic lupus erythematosus patients in our inception cohort show statistical significance in dermatological and vascular manifestations, serositis, granular casts, low kidney glomerular filtration, and high proteinuria, predominantly in male patients. Notably, the renal manifestations did not necessarily lead to a requirement for dialysis or hemodialysis, suggesting that the high frequency of renal disease was not associated with severe renal impairment. Immunological features, particularly positive ds-DNA antibodies, were prevalent in male patients, while females displayed lower hemoglobin levels, indicating the presence of anemia as a suggestive lupus feature in Mexican children.

While our study acknowledges the 10-year difference in inclusion periods for male and female patients as a limitation, it is crucial to emphasize that this is the first investigation of its kind within the Mexican pediatric population. With our study primarily focusing on description, we anticipate that our findings will catalyze future research endeavors involving more temporally aligned cohorts.

Finally, according to our results, the distinct disease characteristics observed in Mexican pediatric patients should be considered in both diagnosis and treatment decisions.
